# Use of Recombinant Human Soluble Thrombomodulin in Patients with Sepsis-Induced Disseminated Intravascular Coagulation after Intestinal Perforation

**DOI:** 10.3389/fmed.2015.00007

**Published:** 2015-02-26

**Authors:** Takashi Tagami, Hiroki Matsui, Kiyohide Fushimi, Hideo Yasunaga

**Affiliations:** ^1^Department of Emergency and Critical Care Medicine, Nippon Medical School, Tokyo, Japan; ^2^Department of Clinical Epidemiology and Health Economics, School of Public Health, Graduate School of Medicine, The University of Tokyo, Tokyo, Japan; ^3^Department of Health Informatics and Policy, Graduate School of Medicine, Tokyo Medical and Dental University, Tokyo, Japan

**Keywords:** anticoagulants, disseminated intravascular coagulation, outcomes assessment, surgery, sepsis

## Abstract

**Background:** Anticoagulant therapy has been evaluated with respect to its potential usefulness in reducing the high mortality rates associated with severe sepsis, including sepsis-induced disseminated intravascular coagulation (DIC) after intestinal perforation. We examined the hypothesis that recombinant human soluble thrombomodulin (rhTM) is effective in the treatment of patients with septic shock with sepsis-induced DIC after laparotomy for intestinal perforation.

**Methods:** We performed propensity-score and instrumental variable analyses of the Japanese Diagnosis Procedure Combination in-patient database, a nationwide administrative database. The main outcome was 28-day in-hospital all-cause mortality.

**Results:** We categorized eligible patients (*n* = 2202) from 622 hospitals into the rhTM group (*n* = 726) and control group (*n* = 1476). Propensity-score matching created 621 matched pairs of patients with and without rhTM. There was neither significant difference in 28-day mortality between the two groups in the unmatched analysis (rhTM vs. control, 25.3 vs. 23.4%, respectively; difference, 1.9%; 95% CI, −1.9 to 5.7) nor in the propensity-score-matched analysis (rhTM vs. control, 26.1 vs. 24.8%, respectively; difference, 1.3%; 95% CI, −3.6 to 6.1). The logistic analysis showed no significant association between the use of rhTM and the mortality in propensity-score-matched patients (OR, 1.1; 95% CI, 0.82–1.4). The instrumental variable analyses, using the hospital rhTM-prescribing proportion as the variable, found that receipt of rhTM was not associated with the reduction in the mortality (risk difference, −6.7%; 95% CI, −16.4 to 3.0).

**Conclusion:** We found no association between administration of rhTM and 28-day mortality in mechanically ventilated patients with septic shock and concurrent DIC after intestinal perforation.

## Introduction

Severe sepsis is one of the most common causes of death in critically ill patients worldwide (1–4). Sepsis-induced disseminated intravascular coagulation (DIC) results in a poor outcome and is frequently observed among these patients (2, 5–7). Sepsis-induced DIC is a systemic process characterized by both thrombosis and hemorrhage (8–12). However, the treatment is generally limited to treatment of the underlying condition and administration of appropriate blood products (9, 11).

Recombinant human soluble thrombomodulin (rhTM; Asahi Kasei Pharma Co., Tokyo, Japan) is used clinically for DIC treatment in Japan (13, 14). Thrombomodulin is a thrombin receptor on the endothelial cell surface and plays a vital part in the regulation of DIC (15, 16). rhTM is composed of the active extracellular domain of thrombomodulin (13). Similar to membrane-bound thrombomodulin, rhTM binds to thrombin to inactivate coagulation, and the thrombin–rhTM complex activates protein C to produce activated protein C (APC). APC inactivates factors VIIIa and Va in the presence of protein S, inhibiting further thrombin formation (13, 16). The clinical impact of rhTM on DIC were first examined in Japan as a phase-3 trial showing that resolution of sepsis-induced DIC was better in patients treated with rhTM than in those treated with heparin (13). Although there was no significant difference in mortality between these two groups of patients (13, 17), the clinical use of rhTM was approved in Japan by the Japanese Ministry of Health, Labour and Welfare in 2008. Vincent et al. (18) recently reported the results of a multinational placebo-controlled trial (phase 2b). Although their study had inadequate power to detect statistical significance, the 28-day mortality rate in patients who received rhTM tended to be lower than that in patients who received standard care alone (17.8 vs. 21.6%, respectively; one-sided log-rank *p* = 0.17) (18). Based on these results, a multinational phase-3 trial of rhTM for sepsis-induced DIC (ClinicalTrials.gov Identifier: NCT01598831) is ongoing.

Previous interventional and epidemiological studies have suggested that the two major infection sites giving rise to sepsis are the lungs and abdomen (1–3). Although the causal pathogens, required interventions, recovery time course, severity of DIC, and mortality rates differ between the lungs and abdomen, infection at these two sites often results in sepsis-induced DIC (1, 6, 19, 20). Additionally, in a previous study of 6342 patients with severe pneumonia and sepsis-induced DIC, we found no significant association between the use of rhTM and the 28-day mortality rate (21). In contrast to our previous study on pneumonia (21), few studies have examined the association between rhTM use and mortality in patients with sepsis-induced DIC with a direct focus on abdominal sepsis and the use of a large sample.

The current study evaluated whether rhTM can reduce mortality in patients with sepsis-induced DIC after intestinal perforation confirmed by laparotomy.

## Materials and Methods

This study was approved by the institutional review board of The University of Tokyo, which waived the requirement for informed patient consent because of the anonymous nature of the data.

### DIC diagnosis and rhTM use in Japan

The Japanese Association for Acute Medicine (JAAM) guidelines were published in 2006 (7). These guidelines have prompted physicians to diagnose sepsis-induced DIC. The guidelines have been widely used in most of the recent reports from Japan (8, 12, 22–24). The JAAM DIC scoring system includes the systemic inflammatory response syndrome score (score of 0 or 1), platelet count (score of 0, 1, or 3), fibrin/fibrinogen degradation product levels (score of 0 or 1), and prothrombin time ratio (score of 0, 1, or 3) (7, 25). DIC was diagnosed in patients with a total score of ≥4 (7). The clinically approved dose of rhTM by the Japanese Ministry of Health, Labour and Welfare is 380 U/kg/day for patients with DIC.

### Data source

For this study, we used the Japanese Diagnosis Procedure Combination (DPC) inpatient database, previously described in detail elsewhere (21, 25–27). In short, this database includes administrative claims and discharge abstract data for all inpatients discharged from more than 1000 participating hospitals, including more than 90% of all tertiary-care hospitals in Japan (21, 25–27). The database contains baseline patient information at admission (day 0), such as age, sex, and consciousness level [Japan Coma Scale score: 0 (alert), 1–3 (delirious), 10–30 (somnolent), and 100–300 (comatose) points (21, 25–27)]. The diagnoses were coded with the International Classification of Diseases 10th Revision codes as main disease, comorbidities at admission, and post-admission complications. Thus, the complications are clearly differentiated from the comorbidities already present in the database. The physicians in charge are obliged to record these diagnoses with reference to the medical charts to optimize the accuracy of the recorded diagnoses. The DPC database also includes the dosages and administration dates of all drugs and blood products administered during hospitalization. All interventional and surgical procedures are also coded with the original codes. The dates with the patients’ status on hospital admission and at discharge are recorded as well (21, 25–27).

### Patient selection

We identified mechanically ventilated patients who required vasopressors and developed DIC after emergency open laparotomy for perforation of the lower intestinal tract as recorded in the DPC database from 1 July 2010 to 31 March 2013. The inclusion criteria were: (1) age of ≥15 years; (2) confirmed diagnosis of perforation of the lower gastrointestinal tract at admission (coded in the “primary diagnosis” or “comorbidities at admission” section of the database); (3) performance of open abdominal laparotomy excluding exploratory laparotomy on day 0 or 1; (4) presence of septic shock, defined as use of vasopressors (noradrenaline and/or dopamine) started on day 0 or 1; (5) performance of mechanical ventilation after surgery on day 0 and/or 1; (6) initiation of antibiotic therapy on day 0 or 1; and (7) diagnosis of DIC (coded in the “main diagnosis” or “comorbidities at admission” section of the database). The exclusion criterion was initiation of rhTM on or after day 2.

### Variables and endpoints

In the current study, the hospital volume was defined as the number of eligible patients treated at each hospital and was subcategorized into tertiles. The hospital type was categorized as academic or non-academic.

The primary endpoint used was 28-day all-cause mortality. The secondary endpoints were ventilator-free days (VFDs) (28) and in-hospital mortality.

### Statistical analysis

We performed propensity-score matching analysis between the rhTM and control groups based on the estimated propensity score (29, 30). The propensity score was estimated using a logistic regression model for receipt of rhTM as a function of the following patient demographics and medication/interventions performed on day 0 or 1: age, gender, hospital type, hospital volume, Japan Coma Scale score, coexisting diseases, performance of blood culture, continuous renal replacement therapy/intermittent hemodialysis, catecholamine use (noradrenaline, dopamine, and/or dobutamine), vasopressin use, steroid use, intravenous immunoglobulin use, heparin use, use of other DIC medications available in Japan (i.e., antithrombin, ulinastatin, nafamostat mesilate, or gabexate mesilate), heparin use, initial use of two or more antibiotics, each types of antibiotics used, selective neutrophil elastase inhibitor use, albumin use, and blood transfusion (1–3, 12, 21, 25–27, 31–34). We regarded the medication/interventions administered simultaneously with rhTM as “pre-rhTM treatments” because the critically ill status requiring these treatments was already present at the time of admission (21). We calculated the *C*-statistic to evaluate the goodness of fit. We performed a one-to-one matched analysis using nearest-neighbor matching based on the estimated propensity scores of the patients. A match occurred when a patient in the rhTM group had an estimated score within 0.2 SD of a patient in the control group (29, 30). We defined absolute values of the standardized difference of more than 10% as out of balance (35), and examined the balance of baseline variables. We compared continuous variables using *t*-tests and categorical variables using the chi-squared test or Fisher’s exact test. We examined the association between administration of rhTM and 28-day mortality using generalized estimating equations (GEE), accounting for the paired nature of the matched patients (36). We also performed Cox regression analysis to assess differences in in-hospital survival rates between patients with and without rhTM in the propensity-score-matched groups.

Instrumental variable analysis was also carried out as a confirmatory analysis of the propensity-score analyses. The hospitals’ preference was used as an instrumental variable, and computed the differences in the 28-day mortality risk between the groups with and without rhTM, using two-stage least-squares method adjusted for the patients’ characteristics (21, 25, 26, 37, 38). We classified hospitals that administered rhTM to the ≥75th percentile of the eligible patients with DIC as hospitals with a preference for rhTM and those that administered rhTM to the <75th percentile of the eligible patients with DIC as hospitals without a preference for rhTM (38). We estimated the risk difference and 95% confidence interval (95% CI) using the *ivreg2* procedure of Stata/SE 13.0 (Stata Corp., College Station, TX, USA). A partial *F*-test was performed to confirm that the patterns of hospital use of rhTM is not a weak instrument (i.e., we examined the null hypothesis that “there was no association between patterns of hospital rhTM use and actual rhTM use”) (37). An *F*-statistic of >10 indicates that the instrument is not weak (37). The IBM SPSS version 22 (IBM, Armonk, NY, USA) and Stata/SE 13.0 were used for all statistical analyses.

## Results

### Patients

**Figure 1:**
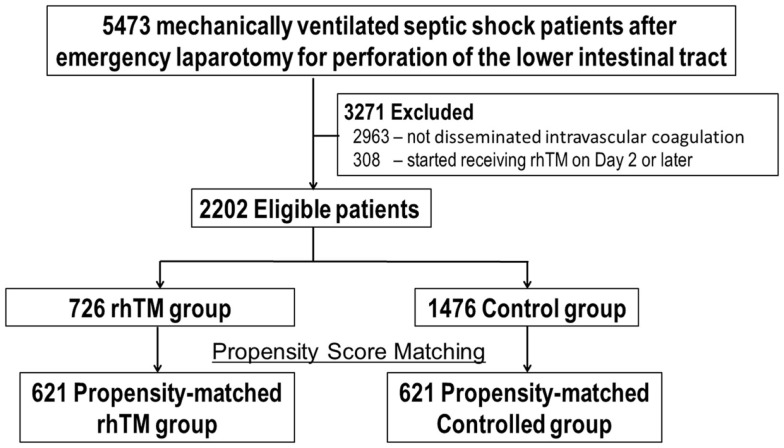
**Eligible patients undergoing propensity-score matching**. DIC, disseminated intravascular coagulation; rhTM, recombinant human soluble thrombomodulin.

Of 5443 mechanically ventilated patients with septic shock after surgically confirmed perforation of the lower intestine, 3271 patients were excluded (2963 due to DIC was not shown and 308 due to delayed start of rhTM). As depicted in Figure 1, 2202 patients (622 hospitals during the 33-month study period) were identified as eligible for the study. These patients were categorized into either an rhTM (*n* = 726) or control (*n* = 1476) group and underwent propensity-score matching, from which 621 propensity-score-matched pairs were generated. The *C*-statistic for the goodness of fit was 0.72 (95% CI, 0.70–0.74) in the propensity-score model. The mean length of hospital stay was 44.8 days (95% CI, 37–52).

Baseline characteristics of the unmatched and propensity-score-matched groups patients were shown in Tables 1 and 2. Patients were more likely to receive rhTM if they were admitted to an academic hospital or required renal replacement therapy, noradrenaline, carbapenem, antithrombin, immunoglobulin, sivelestat sodium, or blood transfusions when the unmatched groups were compared. After propensity-score matching were performed, all of the baseline patient characteristics were well balanced (i.e., the absolute value of the standardized differences <10%) between the groups.

**Table 1:** **Baseline patient characteristics in the unmatched and propensity-score-matched groups**.

	Unmatched groups			Propensity-score-matched groups		
	Control (*n* = 1476)	rhTM (*n* = 726)	Standardized difference, %	Control (*n* = 621)	rhTM (*n* = 621)	Standardized difference, %
**Age (years)**						
18–50	52 (3.5)	18 (2.5)	6.1	13 (2.1)	14 (2.3)	−1.1
51–60	102 (6.9)	41 (5.6)	5.2	35 (5.6)	37 (6.0)	−1.4
61–70	270 (18.3)	142 (19.6)	−3.2	120 (19.3)	112 (18.0)	3.3
71–80	472 (32.0)	234 (32.2)	−0.5	205 (33.0)	203 (32.7)	0.7
≥81	580 (39.3)	291 (40.1)	−1.6	248 (39.9)	255 (41.1)	−2.3
**Sex (male)**	733 (49.7)	354 (48.8)	1.8	310 (49.9)	300 (48.3)	3.2
**Hospital type (academic)**	363 (24.6)	235 (32.4)	−17.3	200 (32.2)	187 (30.1)	4.5
**Hospital volume, cases**						
Low, 1–7	639 (43.3)	271 (37.3)	12.2	242 (39.0)	244 (39.3)	−0.7
Medium, 8–13	398 (27.0)	233 (32.1)	−11.3	184 (29.6)	188 (30.3)	−1.4
High, ≥14	439 (29.7)	222 (30.6)	−1.8	195 (31.4)	189 (30.4)	2.1
**Consciousness level (JCS score)**						
Alert	1003 (68.0)	466 (64.2)	8.0	407 (65.5)	406 (65.4)	0.3
Delirious	248 (16.8)	144 (19.8)	−7.8	109 (17.6)	116 (18.7)	−2.9
Somnolent	99 (6.7)	50 (6.9)	−0.7	39 (6.3)	41 (6.6)	−1.3
Comatose	126 (8.5)	66 (9.1)	−2.0	66 (10.6)	58 (9.3)	4.3
**Coexisting disease**						
Diabetes	140 (9.5)	76 (10.5)	−3.3	72 (11.6)	56 (9.0)	8.5
Old myocardial infarction	20 (1.4)	12 (1.7)	−2.4	10 (1.6)	10 (1.6)	0.0
Chronic obstructive pulmonary disease	44 (3.0)	18 (2.5)	3.1	17 (2.7)	17 (2.7)	0.0
Pneumonia	96 (6.5)	43 (5.9)	2.4	36 (5.8)	39 (6.3)	−2.0
Liver cirrhosis	19 (1.3)	2 (0.3)	11.5	2 (0.3)	2 (0.3)	0.0
Chronic renal failure	111 (7.5)	51 (7.0)	1.9	49 (7.9)	46 (7.4)	1.8

**Table 2:** **Medications and interventions performed on day 0 or 1 in the unmatched and propensity-score-matched groups**.

	Unmatched groups			Propensity-score-matched groups		
	Control (*n* = 1476)	rhTM (*n* = 726)	Standardized difference, %	Control (*n* = 621)	rhTM (*n* = 621)	Standardized difference, %
**Blood culture taken**	635 (43.0)	377 (51.9)	−17.9	317 (51.0)	310 (49.9)	2.3
**Intermittent hemodialysis**	237 (16.1)	126 (17.4)	−3.5	107 (17.2)	104 (16.7)	1.3
**Continuous renal replacement therapy**	389 (26.4)	269 (37.1)	−23.1	207 (33.3)	213 (34.3)	−2.0
**Polymyxin B hemoperfusion**	403 (27.3)	226 (31.1)	−8.4	182 (29.3)	186 (30.0)	−1.4
**Catecholamines**						
Dopamine use	1205 (81.6)	580 (79.9)	4.4	501 (80.7)	504 (81.2)	−1.2
Noradrenaline use	993 (67.3)	557 (76.7)	−21.2	464 (74.7)	462 (74.4)	0.7
Dobutamine use	210 (14.2)	148 (20.4)	−16.3	100 (16.1)	114 (18.4)	−6.0
**Vasopressin**	84 (5.7)	65 (9.0)	−12.5	45 (7.2)	49 (7.9)	−2.4
**Initial antibiotic use**						
Initial use of two or more antibiotics	321 (21.7)	175 (24.1)	−5.6	145 (23.3)	149 (24.0)	−1.5
Pazobactam/piperacillin or sulbactam/cefoperazone sodium	117 (7.9)	70 (9.6)	−6.1	52 (8.4)	60 (9.7)	−4.5
First-generation cephalosporin	52 (3.5)	30 (4.1)	−3.2	26 (4.2)	27 (4.3)	−0.8
Second-generation cephalosporin	511 (34.6)	206 (28.4)	13.5	187 (30.1)	185 (29.8)	0.7
Third-generation cephalosporin	32 (2.2)	16 (2.2)	−0.2	13 (2.1)	16 (2.6)	−3.2
Fourth-generation cephalosporin	44 (3.0)	19 (2.6)	2.2	16 (2.6)	18 (2.9)	−2.0
Carbapenem	971 (65.8)	531 (73.1)	−16.0	446 (71.8)	439 (70.7)	2.5
**Antifungal drug**	29 (2.0)	20 (2.8)	−5.2	14 (2.3)	15 (2.4)	−1.1
**Low-dose steroid**	158 (10.7)	78 (10.7)	−0.1	57 (9.2)	65 (10.5)	−4.3
**Antithrombin**	622 (42.1)	439 (60.5)	−37.3	350 (56.4)	347 (55.9)	1.0
**Gabexate mesilate**	670 (45.4)	162 (22.3)	50.3	148 (23.8)	161 (25.9)	−4.8
**Nafamostat mesilate**	826 (56.0)	457 (62.9)	−14.3	375 (60.4)	380 (61.2)	−1.6
**Ulinastatin**	497 (33.7)	218 (30.0)	7.8	187 (30.1)	190 (30.6)	−1.1
**Heparin**	1228 (83.2)	614 (84.6)	−3.7	524 (84.4)	522 (84.1)	0.9
**Immunoglobulin**	740 (50.1)	419 (57.7)	−15.2	345 (55.6)	347 (55.9)	−0.6
**Sivelestat sodium**	680 (46.1)	394 (54.3)	−16.5	308 (49.6)	330 (53.1)	−7.1
**Albumin**	1222 (82.8)	630 (86.8)	−11.1	529 (85.2)	534 (86.0)	−2.3
**Blood transfusion**						
Red blood cells	844 (57.2)	470 (64.7)	−15.5	389 (62.6)	391 (63.0)	−0.7
Fresh frozen plasma	934 (63.3)	456 (62.8)	1.0	379 (61.0)	392 (63.1)	−4.3
Platelets	230 (15.6)	150 (20.7)	−13.2	113 (18.2)	116 (18.7)	−1.2
**Volume of first blood transfusion**						
Overall no. of transfusions	556 (37.7)	217 (29.9)	16.5	206 (33.2)	199 (32.0)	2.4
≤500 mL	355 (24.1)	191 (26.3)	−5.2	161 (25.9)	161 (25.9)	0.0
501–1000 mL	413 (28.0)	232 (32.0)	−8.7	177 (28.5)	190 (30.6)	−4.6
≥1001 mL	152 (10.3)	86 (11.8)	−4.9	77 (12.4)	71 (11.4)	3.0

*rhTM, recombinant human soluble thrombomodulin*.

### Endpoint

The overall 28-day mortality rate was 24.1% (530/2202). No significant difference was documented in the 28-day mortality rate between the two groups in the unmatched analysis [rhTM vs. control, 25.3% (184/726) vs. 23.4% (346/1476), respectively; difference, 1.9%; 95% CI, −1.9 to 5.7] or in the propensity-score-matched analysis [rhTM vs. control, 26.1% (162/621) vs. 24.8% (154/621), respectively; difference, 1.3%; 95% CI, −3.6 to 6.1]. The logistic GEE analysis showed no significant association between the use of rhTM and 28-day mortality in propensity-score-matched patients (OR, 1.1; 95% CI, 0.82-1.4). In the instrumental variable model, the null hypothesis (i.e., no association between hospital rhTM use and actual rhTM use) was rejected with an *F*-statistic of 370 (i.e., *F*-statistic >10, *p* < 0.001). The reduction in the 28-day mortality was not significantly associated with receipt of rhTM (risk difference, −6.7%; 95% CI, −16.4 to 3.0).

We found no significant difference in the in-hospital mortality between the two groups of unmatched patients [rhTM vs. control, 35.7% (259/726) vs. 33.1% (489/1476), respectively; difference, 2.5%; 95% CI, −1.7 to 6.8]. Moreover, no significant difference was found among the propensity-score-matched patients [rhTM vs. control, 35.9% (223/621) vs. 34.3% (213/621), respectively; difference, 3.2%; 95% CI, −3.7 to 6.9]. Cox regression analysis showed no significant difference in in-hospital mortality between the two groups (hazard ratio, 1.1; 95% CI, 0.88–1.3).

We found no significant difference in the number of VFDs between the rhTM group and control groups among either unmatched patients (14.0 vs. 14.7 days, respectively; difference, −0.7; 95% CI, −1.7 to 0.3) or propensity-score-matched patients (14.2 vs. 14.3 days, respectively; difference, −0.17; 95% CI, −1.0 to 1.4).

## Discussion

The current study suggests that there is no significant association between rhTM use and 28-day mortality in sepsis-induced DIC patients after laparotomy for intestinal perforation. Additionally, no significant difference was documented in the VFDs between the rhTM and control groups.

Anticoagulant therapy agents, such as antithrombin (39–41) and recombinant APC (42), have been evaluated as additional therapies to reduce the high mortality rates associated with severe sepsis and septic shock. Antithrombin supplementation as an adjunct therapy in patients with sepsis-induced DIC was suggested in the 1990s (40, 41). However, the largest randomized trial, the KyberSept Trial (43), could not prove beneficial effects of high-dose antithrombin therapy on mortality. Moreover, meta-analyses of randomized trials concluded that antithrombin could not be generally recommended for critically ill patients, including those with severe sepsis (44). Conversely, Bernard et al. (42) suggested the efficacy of APC on survival in patients with severe sepsis in 2001. However, APC was withdrawn from the market after the Prospective Recombinant Human APC Worldwide Evaluation in Severe Sepsis and Septic Shock trial documented its failure (45). Thus, the latest Surviving Sepsis Campaign Guidelines (3), the major international guidelines for severe sepsis, do not recommend the use of antithrombin and APC for patients with severe sepsis.

A novel anticoagulant therapy, rhTM, was developed for patients with DIC in Japan. Several studies have investigated the use of rhTM for sepsis-induced DIC in Japan, which is the only country where rhTM is widely used for clinical treatment of DIC (46–51). Several positive effects of rhTM were reported in these studies (46–51). Additionally, Vincent et al. (18) reported that rhTM was associated with beneficial pharmacologic effects and is a safe intervention in critically ill patients with sepsis-induced coagulopathy. Thus, rhTM seems to be a promising drug for sepsis-induced DIC.

Previous studies have suggested that patients with respiratory and/or cardiac dysfunction and evidence of DIC appear to receive the greatest rhTM-related survival benefit (13, 17, 18, 47, 51, 52). Therefore, in the current study, we selected patients with DIC who required vasopressor and mechanical ventilation after emergency open laparotomy for perforation of the lower intestinal tract. The strength of the current study is the evaluation of more than 2000 patients using data throughout Japan. We used a propensity-score matching approach to overcome the bias associated with the measured confounding factors. After propensity-score matching, the selected patients were well balanced with regard to baseline characteristics, including factors with the potential to affect mortality in patients with septic shock after emergency laparotomy, as presented in Tables 1 and 2. We then attempted to construct a “randomized experiment-like situation,” and compared groups with similar characteristics and conditions without specifying the relationships between measured confounding factors and outcomes. However, we failed to confirm our hypothesis that rhTM is associated with better survival and with reducing VFDs in the propensity-score-matched analyses. Recent studies indicated that rhTM acts as an anticoagulant with anti-inflammatory properties that may prevent later occurrence of multiple organ failure (12, 49, 53). However, we could not demonstrate this effect when analyzing our data. Although the number of VFDs should have represented the recovery of pulmonary failure in the patients in the current study, there was no significant difference in the number of VFDs between the rhTM and control groups. These findings are consistent with the results obtained by logistic GEE and Cox regression analyses. Moreover, we performed also instrumental variable analysis to address the bias of confounding factors that were not unmeasured, and the outcome of this analysis confirmed the robustness of the results.

The current results are consistent with those of two randomized trials in patients with sepsis-induced DIC (13, 18) as well as with our previous large retrospective study that evaluated patients with severe pneumonia with sepsis-induced DIC (21), all of which reported non-significant mortality trends in favor of rhTM. However, we could not obtain any objective coagulation status data from the database, including platelet counts, D-dimer or fibrinogen levels, and DIC scores. The scoring systems for DIC, which exhibit various differences, were used as an inclusion criterion and quantitatively evaluated in previous studies (13, 17, 18, 51). Thus, we must wait for the results of an ongoing multinational phase-3 trial (ClinicalTrials.gov Identifier: NCT01598831) before drawing any further conclusions.

There are some limitations in the current study. First, our study was performed retrospectively without randomization. Although a propensity-score method was used to adjust for measured confounding factors, bias could inevitably exist in the form of confounders that were not measured. For example, although approximately 20% of patients were administered dobutamine, the necessary information for calculating cardiac index of the patients was not available in the database. More importantly, DIC scores, which may indicate the severity of DIC, were also unavailable. We therefore performed instrumental variable analyses to compensate for these potentially unmeasured confounders. Second, the definition of septic shock in the current study might be vague. Vasopressors might be used for types of shock other than septic shock. Additionally, according to the Surviving Sepsis Campaign 2012 (3), septic shock is defined as “sepsis-induced hypotension persisting despite adequate fluid resuscitation.” In the current study, we used a surrogate definition because the term “adequate” could not be accurately evaluated from the information in the database. However, we believe that if the physician in charge determined that fluid resuscitation was not “adequate” in patients with septic shock, the next step for resuscitation must be the use of vasopressors according to the guidelines. This explains the definition of septic shock used in the current study. Third, we could not determine the exact time when rhTM was administered. We therefore could not identify if the variables used in the propensity-score estimation were pre- or post-rhTM treatment. In the latter case, those variables could not be used for estimating the propensity-score (21, 54, 55). However, rhTM has a long half-life of 20 h. Therefore, rhTM could not have affected the other interventions/medications within days 0 and 1 (12, 13, 17, 21, 56–58). Fourth, we only evaluated mechanically ventilated patients with concurrent septic shock and DIC after surgery for intestinal perforation. As a result, only a few patients in this strictly selected population were included from each hospital. Because they were from various institutions, they might not have been uniformly monitored and treated. Additionally, the results cannot be generalized to patients with other causes of abdominal sepsis such as cholangitis, cholecystitis, and pancreatitis. Fifth, the selective use of concurrent dugs affecting coagulation, including antithrombin, gabexate mesilate, nafamostat mesilate, and heparin, may have affected the results of the study, although the proportions of these drugs were well balanced between the rhTM and control groups after propensity-score matching. These drugs might be used for the treatment of a variety of conditions in critical care other than DIC therapy (e.g., treatment of pancreatitis or anticoagulation for hemodialysis and/or placement of an arterial line).

## Conclusion

We found no association between administration of rhTM and 28-day mortality in mechanically ventilated patients with septic shock and concurrent DIC after intestinal perforation. Prospective randomized trials are required to confirm our findings.

## Author Contributions

TT contributed to study design, statistical analyses, and review and drafting of the manuscript. HM contributed to study design, data acquisition, statistical analyses, and review of the manuscript. KF contributed to study design, data acquisition, and review of the manuscript. HY contributed to study design, statistical analyses, data acquisition, and review and drafting of the manuscript. All authors read and approved the final version of the manuscript.

## Conflict of Interest Statement

The authors declare that the research was conducted in the absence of any commercial or financial relationships that could be construed as a potential conflict of interest.
